# Non-Stick Liver Parenchymal Transection With Saline-Linked Bipolar Clamp-Crush Technique in Robotic Liver Resection

**DOI:** 10.7759/cureus.36401

**Published:** 2023-03-20

**Authors:** Masatoshi Kajiwara, Takahisa Fujikawa, Shigetoshi Naito, Takahide Sasaki, Ryo Nakashima, Suguru Hasegawa

**Affiliations:** 1 Gastroenterological Surgery, Faculty of Medicine, Fukuoka University, Fukuoka, JPN; 2 Surgery, Kokura Memorial Hospital, Kitakyushu, JPN

**Keywords:** clamp-crush technique, saline drip, saline-linked, bipolar forceps, robotic liver resection, liver parenchymal transection, liver surgery

## Abstract

Background

Without satisfactory instruments, liver parenchymal transection during robotic liver resection (RLR) remains challenging. We combined the commonly used bipolar clamp-crush technique with the saline drip, achieving a comfortable liver resection without coagulated liver tissues sticking to the bipolar forceps.

Methods

Between December 2022 and March 2023, six RLRs were performed using the saline-linked bipolar clamp-crush method for both anatomical and non-anatomical liver resections. We assessed the safety and feasibility of our robotic liver parenchymal transection technique.

Results

Three of six patients were diagnosed with colorectal liver metastasis, two with hepatocellular carcinoma (HCC), and the other with intrahepatic bile duct stricture. Three of the six patients received anatomical liver resection, and the other three underwent non-anatomical liver resection. There were no conversions to open surgery. The median operative time and estimated blood loss were 406.5 minutes (196-670 minutes) and 5 ml (5-465 ml), respectively. The median length of the postoperative hospital stay was nine days (7-10 days). Postoperative complications (Clavien-Dindo classification grade II or more) or mortality were not encountered in this cohort.

Conclusion

We presented here our saline-linked bipolar clamp-crush method for liver parenchymal transection in RLR. By simply adding the saline drip to the commonly used bipolar clamp-crush technique, non-stick and comfortable liver parenchymal transection is now possible. This technique may help overcome the limitations of currently available robotic instruments for liver parenchymal resection.

## Introduction

Hepatectomy is increasingly performed via a robotic approach, like other gastrointestinal surgeries [1-4]. In open or laparoscopic liver resection, various optimized devices for parenchymal dissection (e.g., ultrasonic aspirator [5,6] and ultrasonic incision coagulator [7,8]) are available. On the other hand, in robotic liver resection (RLR), there is no forceps suitable for liver parenchymal resection, which is a major problem in RLR.

Consequently, the old-fashioned clamp-crush technique is getting attention again [9]. However, one of the disadvantages of the clamp-crush technique is that the coagulated or necrotic liver tissues stick to the tip of the forceps when the bipolar energy of the Maryland dissector is activated. Withdrawing and inserting the instruments for cleaning multiple times results in a non-negligible loss of operation time, especially in robotic surgery.

We present here a comfortable liver resection method free from the stress of eschar and char formation on the bipolar forceps by combining the traditional clamp-crush technique with the saline drip in RLR.

## Materials and methods

Patients and methods

From December 2022 to March 2023, six cases of robotic liver resections (RLRs) were performed using the saline-linked bipolar clamp-crush method at Fukuoka University Hospital, Japan. Perioperative parameters such as the patient’s age, gender, body mass index (BMI), pathologic condition, tumor size, operative procedure, operative time, estimated blood loss, and length of postoperative hospital stay were retrospectively obtained from the medical records. All continuous variables were expressed as medians with ranges. Postopearative complications were expressed using the Clavien-Dindo Classification [10]. This study was approved by the institutional review board of Fukuoka University Hospital (H22-03-002). Because of the retrospective study design, informed consent was substituted for an informed opt-out procedure, and anonymized data were used.

Surgical technique

**Figure 1:**
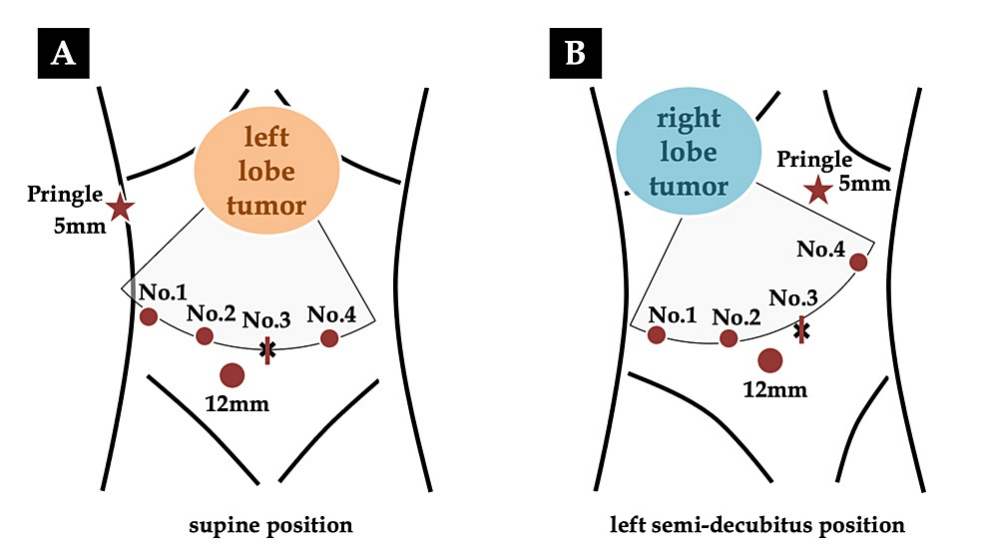
Trocar placement of robotic liver resection for left lobe tumors (A) and right lobe tumors (B) Four robotic trocars were positioned concentrically around the targeted liver lobe. (The figure is the authors' own creation.)

The Da Vinci Xi system was used to perform all six RLRs in this study. The detailed settings of RLR were described previously [11]. Briefly, for left lobe tumors, the patient was placed supine in a 10° reverse Trendelenburg position with legs apart, and for right lobe tumors, the patient was placed in a left semi-decubitus position with a 10° head up. An incision on the top of the navel was made to insert the first robotic trocar (No. 3). Pneumoperitoneum was created, and intraabdominal pressure was maintained between 8 and 10 mmHg. The other three robotic trocars were inserted concentrically around the targeted liver lobe with a 12-mm trocar for the assistant surgeon, which was used for saline dripping, as will be described below, and stapling of Glissonean pedicles or hepatic veins (Figure 1).

The extraperitoneal tourniquet system for intermittent vascular occlusion of the hepatoduodenal ligament (Pringle maneuver) was also placed, as shown in Figure 1 [12]. The Pringle maneuver was routinely applied during liver parenchymal transection.

For left lobe tumors, No. 1, 2, 3, and 4 robotic trocars were assigned to liver retraction (Tip-Up Forceps®), the operator’s left hand (EndoWrist Suction Irrigator®), the 30° endoscope, and the right hand (Maryland Bipolar Forceps®), respectively. For right lobe tumors, No. 1, 2, 3, and 4 robotic trocars were used for the operator’s left hand (EndoWrist Suction Irrigator®), the 30° endoscope, the right hand (Maryland Bipolar Forceps®), and liver retraction (Tip-Up Forceps®), respectively. 

**Figure 2:**
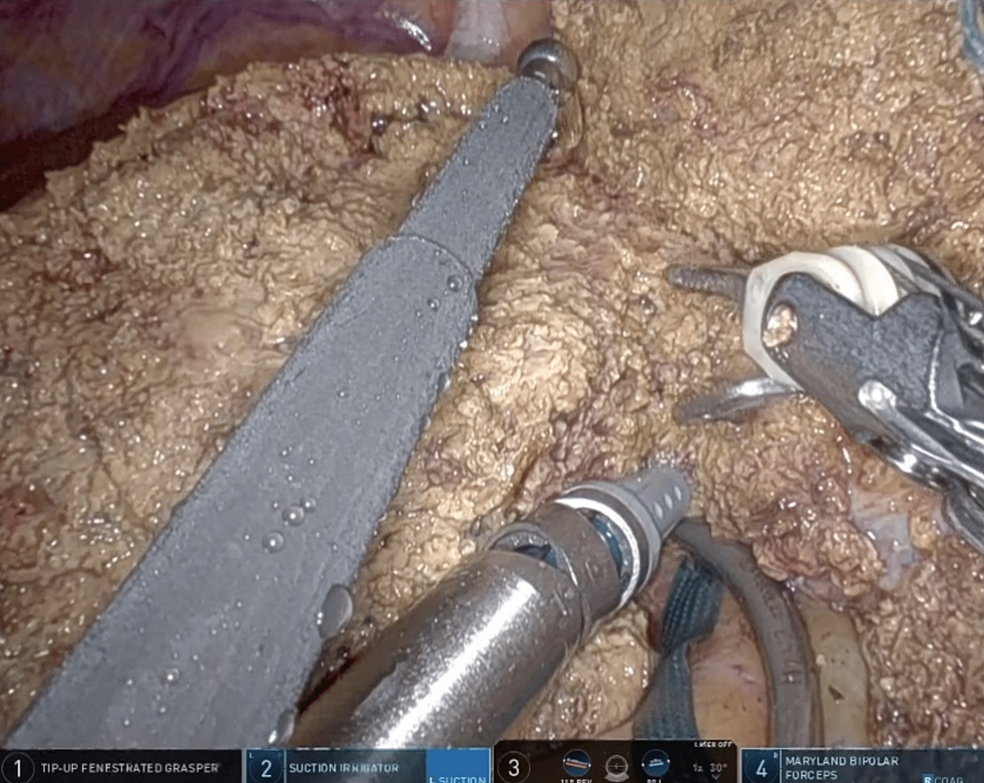
Saline-linked bipolar clamp-crush method The saline-linked bipolar clamp-crush method using Maryland Bipolar Forceps® and EndoWrist Suction Irrigator® with the ball-tipped electrocautery dripping the saline. (Left hemihepatectomy [Case 5])

The assistant surgeon continuously dripped a saline solution (the drip rate was set at 1-2 cc/minute) onto the Maryland Bipolar Forceps® using the ball-tipped electrocautery during the clamp-crush method for liver parenchymal transection (Figure 2 and Video 1).

**Video 1:** Saline-linked bipolar clamp-crush method The saline-linked bipolar clamp-crush method using Maryland Bipolar Forceps® and EndoWrist Suction Irrigator® with the ball-tipped electrocautery dripping the saline. (Left hemihepatectomy [Case 5])

The clamp-crush method starts by gently clamping and crushing the liver parenchyma with the forceps. If there are no apparent vessels or only small vessels, the bipolar energy is turned on, and tissue dissection is performed. Vascular structures with a diameter greater than 2 mm are separated by ultrasonic coagulating shears or after clipping.

## Results

The background characteristics of six cases are shown in Table 1.

**Table 1:** Patients' background characteristics BMI: body mass index, HCC: hepatocellular carcinoma

Case No.	Age (years)	Sex	BMI (kg/m^2^)	Pathologic condition	Maximum tumor diameter (mm)	Liver cirrhosis	Preoperative chemotherapy
1	57	M	28.5	colorectal liver metastasis	15	No	No
2	65	F	22.1	colorectal liver metastasis	12	No	Yes
3	53	M	26.3	colorectal liver metastasis	8	No	No
4	71	M	20.2	HCC	35	No	No
5	79	F	22.8	bile duct stricture	-	No	No
6	70	M	15.1	HCC	70	Yes	No

The median age of the six patients was 67.5 years (53-79 years). Three of six patients were diagnosed with colorectal liver metastasis, two with hepatocellular carcinoma (HCC), and the other with intrahepatic bile duct stricture. The median maximum tumor diameter was 15 mm (8-70 mm). One patient had liver cirrhosis, and another patient had preoperative chemotherapy for multiple colorectal liver metastases. Table 2 shows the surgical procedures and outcomes in these cohorts.

**Table 2:** Surgical procedures and outcomes of the enrolled patients

Case No.	Procedure	Operative time (min)	Estimated blood loss (ml)	Postoperative hospital stay (days)	Postoperative complication	Mimimum surgical margin (mm)
1	partial resection	196	5	9	none	10
2	partial resection x4	670	465	7	none	1
3	partial resection x5	289	5	9	none	1
4	left hemihepatectomy	472	148	8	none	1
5	left hemihepatectomy	341	5	9	none	10
6	S7 segmentectomy	487	5	10	none	10

Six RLRs consisted of three anatomical resections (left hemihepatectomy [n=2] and S7 segmentectomy [n=1]) and three non-anatomical resections (single partial resection [n=1] and multiple partial resections [n=2]). There were no conversions to open surgery. The median operative time and estimated blood loss were 406.5 minutes (196-670 minutes) and 5 ml (5-465 ml), respectively. Postoperative complications (Clavien-Dindo classification grade II or more) or mortality did not occur. The median length of the postoperative hospital stay was nine days (7-10 days). Negative surgical margins were achieved in all six cases.

## Discussion

This pilot series showed that the simple method combining the conventional bipolar clamp-crush technique with the saline drip was able to eliminate necrotic or coagulated liver tissue formation on the bipolar forceps. 

Several robotic liver transection methods have been introduced so far [1,13-15], but eschar or char formation on the instruments has not been described in detail yet and has not been resolved. With the conventional clamp-crush method, liver transection can be performed smoothly in a bloodless environment. However, once bleeding occurs and the bipolar forceps are applied to the bleeding site, coagulated liver tissues often stick to the bipolar, making hemostasis more difficult, creating a vicious cycle.

As a novel technique to prevent this eschar or char formation on the robotic instruments, we recently reported the "Saline-linked Monopolar Cautery Scissors (SLiC-Scissors)" method [11], based on the "Kyoto University-style liver parenchymal transection" procedure [16], and presented a better outcome. However, in order to transect the liver parenchyma with spatula-shaped scissors and control the bleeding in all places, there are some knacks, such as the appropriate angle of the scissors and the proper pressure at the bleeding site, and it takes time to get used to them. In addition, since the scissors are monopolar electrodes, it is necessary to use other devices, such as a waterjet scalpel, to avoid heat damage when dissecting around the Glissonean pedicle.

Given this, we introduced the combination of the traditional bipolar clamp-crush technique and the saline drip as a simpler and easier method while maintaining the concept of the saline drip, which is the leading role of the non-sticking "SLiC-Scissors" technique for RLR [11].

As a result, necrotic or coagulated liver tissue formation on the bipolar forceps was effectively prevented as expected, and cleaning of the bipolar due to severe eschar or char formation was not required during liver parenchymal transection when appropriate moisture is ensured. In addition, the clamp-crush method has been often used for open or laparoscopic hepatectomy so far, and there has been no stress or restriction on how to use the wrist to move the bipolar forceps.

If the amount of saline in the operative field is low, eschar and char formation will occur. On the other hand, excess saline solution will interfere with the bipolar forceps turning on electricity, meaning that an appropriate moisture level is quite essential as described in the “SLiC-Scissors” method [11]. Therefore, it is important to request the assistant surgeon to drip sufficient saline and finely adjust the moisture with the suction on the operator's left hand. As the suction of the Da Vinci Xi system is equipped with EndoWrist® function, it is also suitable for supporting a stable liver transection plane at the same time as suctioning.

Although an assistant surgeon is required to make the saline drip follow the bipolar forceps using the ball-tipped electrocautery, it seems to be a benefit that the ball-tipped electrode (in soft coagulation mode) can reach the bleeding point immediately in the event of bleeding that is uncontrollable with the bipolar forceps.

The drawback of the present report is that this is a pilot study with a small sample size. In order to provide substantial evidence, it is necessary to apply this method to more cases in the future and further confirm its usefulness.

## Conclusions

We presented here our saline-linked bipolar clamp-crush method for liver parenchymal transection in RLR. By simply adding the saline drip to the commonly used bipolar clamp-crush technique, non-stick and comfortable liver parenchymal transection is now possible. It is quite essential to maintain appropriate moisture in the liver transection plane by dripping satisfactory saline and intermittent suctioning. This safe and feasible technique helps overcome the limitations of currently available robotic instruments for liver parenchymal resection and is expected to lead to the standardization of RLR.
